# Peptide-Decorated Microneedles for the Detection of Microplastics

**DOI:** 10.3390/bios14030140

**Published:** 2024-03-12

**Authors:** Suyeon Ahn, Namju Kim, Yonghyun Choi, Jiwon Kim, Hyeryun Hwang, Cholong Kim, Hee-Young Lee, Seungyoun Kim, Jin Su Kim, Hyun Ho Lee, Jonghoon Choi

**Affiliations:** 1 School of Integrative Engineering, Chung-Ang University, Seoul 06974, Republic of Korea; tndus971@naver.com (S.A.); knj9769@cau.ac.kr (N.K.); dydgus5057@cau.ac.kr (Y.C.); jacqueline46@cau.ac.kr (J.K.); 2 Feynman Institute of Technology, Nanomedicine Corporation, Seoul 06974, Republic of Korea; 3 Department of Chemical Engineering, Myongji University, Yongin-si 17058, Republic of Korea; gpduswldus@naver.com (H.H.); rlachfhd2386@naver.com (C.K.); 4 Department of Chemical Engineering, Kumoh National Institute of Technology, Gumi-si 39177, Republic of Korea; lhysshr@kumoh.ac.kr; 5 Division of Applied RI, Korea Institute of Radiological and Medical Sciences (KIRAMS), Seoul 01812, Republic of Korea; sn6704@kirams.re.kr

**Keywords:** microplastic, peptide, microneedle, Raman spectroscopy

## Abstract

The escalating utilization of plastics in daily life has resulted in pervasive environmental pollution and consequent health hazards. The challenge of detecting and capturing microplastics, which are imperceptible to the naked eye, is exacerbated by their diminutive size, hydrophobic surface properties, and capacity to absorb organic compounds. This study focuses on the application of peptides, constituted of specific amino acid sequences, and microneedles for the rapid and selective identification of microplastics. Peptides, due to their smaller size and greater environmental stability compared with antibodies, emerge as a potent solution to overcome the limitations inherent in existing detection methodologies. To immobilize peptides onto microneedles, this study employed microneedles embedded with gold nanorods, augmenting them with sulfhydryl (SH) groups at the peptides’ termini. The sensor developed through this methodology exhibited efficient peptide binding to the microneedle tips, thereby facilitating the capture of microplastics. Raman spectroscopy was employed for the detection of microplastics, with the results demonstrating successful attachment to the microneedles. This novel approach not only facilitates localized analysis but also presents a viable strategy for the detection of microplastics across diverse environmental settings.

## 1. Introduction

Microplastics, defined as synthetic organic polymers smaller than 5 mm [1], have emerged as a significant environmental pollutant, largely attributable to their extensive application in everyday products due to their malleability and color variability [2]. The recent COVID-19 pandemic has precipitated a surge in the utilization of plastic-based items such as masks, disposable packaging, and medical supplies [3,4], exacerbating the volume of plastics relegated to waste. Given the non-biodegradable nature of most plastics, which may persist for centuries before natural decomposition [5], the escalation in consumption and emissions, alongside overproduction, indiscriminate use, limited recycling efforts, and inadequate regulatory measures to curb waste [6], have significantly contributed to environmental pollution and microplastic generation. The omnipresence of microplastics across various industries, and their potential for human exposure through inhalation and ingestion, raise substantial health concerns [7], necessitating research into their detection. Unlike larger plastic debris, microplastics’ diminutive size renders them invisible to the naked eye, with further complications in their detection and analysis due to the diversity in polymer morphologies and properties [8]. Identifying the presence of microplastics involves quantifying their abundance and analyzing their chemical composition [9]. Techniques such as microscopy, pyrolysis–gas chromatography, Fourier transform infrared (FT-IR) spectroscopy, and Raman spectroscopy have been applied to this end [10,11]. In particular, Raman spectroscopy has demonstrated efficiency in detecting microplastics of minimal dimensions [12]. This technique distinguishes samples with high specificity by identifying molecular vibrations, which serve as chemical structure fingerprints within the Raman spectrum [13]. Common plastic polymers such as polystyrene (PS), polyethylene (PE), and polypropylene (PP) each have a unique carbonaceous backbone structure [14]. For example, PS is a polymer that contains phenyl groups, and these structures can be observed in Raman analysis as specific vibrational frequency patterns. In addition, Raman analysis is based on light scattering, so when detecting a light source under laser illumination, information about the molecule’s vibrations can be obtained without it absorbing light, regardless of the surface morphology or structure of the sample [15]. This means that it can provide more reliable results than FT-IR analysis for samples with irregular surfaces, such as microplastics. The non-destructive nature of Raman spectroscopy, along with its minimal sample requirements and rapid analysis capability, highlights its utility in microplastic analysis [16]. However, effective sample collection, a critical precursor to analysis, is often challenged by microplastics’ hydrophobic surfaces and their tendency to adsorb various organic substances [17].

**Figure 1:**
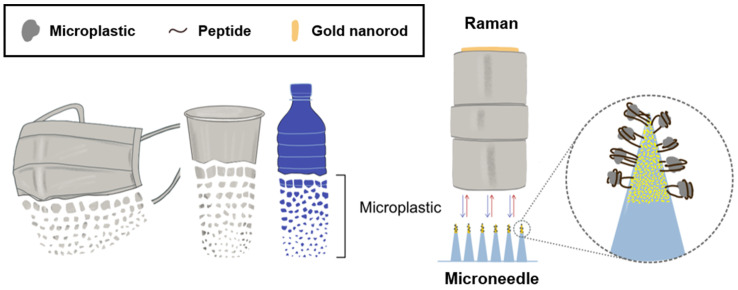
Illustration of microplastics detection using peptides in microneedles containing gold nanorods.

In the preceding research, we have identified microplastics using peptides that exhibit specific binding affinities for particular plastic types [18]. These peptides demonstrated a high affinity for both unoxidized and oxidized forms of PS and PP [18]. Aiming to enhance the specificity and simplicity of microplastic capture, we explored the use of microneedles. By confirming the binding of microplastics—specifically smaller-sized PS, PP, and PE—to targeted peptides, we designed a microneedle tip integrated with gold nanorods. Gold (Au) is well known for its ability to easily combine with Au and sulfur (S) [19]. In addition, the morphology of gold nanorods is an asymmetric or aspherical structure that can exhibit maximum plasmonic effects such as surface-enhanced Raman spectra (SERS), which is the main reason for using Au. These nanorods were modified with peptides terminated with sulfhydryl (SH) groups to selectively bind microplastics, a process subsequently analyzed via Raman spectroscopy (Figure 1). Our findings herald a significant advancement in the selective and straightforward capture of microplastics in environmental settings.

## 2. Materials and Methods

### 2.1. Materials

PS (average molecular weight: 35,000), PP (average molecular weight: ~12,000), and PE (average molecular weight: ~4000) were procured from Sigma-Aldrich (St. Louis, MO, USA). PS microspheres (part number: PSMS-1.07, diameter 9.5–11.5 mm) and PE microspheres (UVPMS-BG-1.00) were purchased from Cospheric (Santa Barbara, CA, USA). SpectraPor^®^ 3.5 kDa dialysis bags, hydrochloric acid (Catalog number H1758), and Tween-80 (Catalog number 28329) were also purchased from Sigma-Aldrich. Peptides designed to bind to plain plastics, specifically HWGMWSY (polystyrene binding peptide; PSBP), MPAVMSSAQVPR (polypropylene binding peptide; PPBP), and LPPWKHKTSGVA (polyethylene binding peptide; PEBP), and peptides terminally modified with fluorescein isothiocyanate (FITC) or 3-Mercaptopropanal groups were sourced from Anygen (Buk-gu, Gwangju, Republic of Korea). The feeding needles (Catalog number JD-S-124) were acquired from JEUNG DO B&P (Nowon-gu, Seoul, Republic of Korea). The 50 mL tube-top vacuum filter system was purchased from Corning (New York, NY, USA), and 30% hydrogen peroxide was purchased from DAEJUNG (Siheung, Republic of Korea). Water, utilized for combining the peptides with the plastics, was purified using a Millipore Milli-Q system (18.2 MU cm resistance).

### 2.2. Preparation of Microplastics

To prepare the microplastics, PS, PP, and PE were first reduced to small fragments using a blender (model UNB-A9100, UNIX, Seoul, Republic of Korea). Following pulverization, the plastic fragments were sorted by size utilizing a series of testing sieves (Chunggye sieve; Gunpo-si, Gyeonggi-do, Republic of Korea) with mesh apertures of 106, 75, 53, and 38 μm. To ensure the precision of the size-based separation, the samples were examined under a microscope subsequent to blending. The size distribution of the resultant microplastics was thereafter quantitatively analyzed and depicted through a size distribution diagram generated using ImageJ (v. 1.54) software.

### 2.3. Fabrication of Microneedle

The PDMS substrate was cured by mixing prepolymer A (Silgard 189, Dupont, Wilmington, DE, USA) with prepolymer B in a volumetric ratio of 10:1. The mixture was thoroughly blended and subsequently degassed under vacuum to eliminate any air bubbles. For the fabrication of a pyramidal microneedle array, a commercially available polycarbonate (PC)-based microneedle array (Smicna Pte Ltd., Singapore) was utilized as the master template to create the PDMS mold [20] The PDMS mold was cleansed thrice using acetone and deionized (DI) water to remove any contaminants. Following this, a prepolymer of NOA 63 was drop-casted onto the engraved PDMS mold. To ensure the complete filling of the NOA 63 prepolymer solution into the mold’s cavities, a vacuum was applied. During this process, the NOA 63 solution might temporarily overflow and form bubbles within the mold. However, the PDMS mold is capable of being used for stamping more than 10 times without experiencing mechanical degradation. Once all bubbles were eliminated from the mold, the NOA 63 moiety was transferred onto a bare glass substrate by stamping or imprinting, and UV light was applied from the underside of the glass substrate for 25 min to cure the resin. The assembly was subsequently heated on a hot plate at 70 °C for 10 min to fully solidify the transparent microneedle array.

In the initial step of filling the PDMS mold, gold nanorods (Au NRs) were applied to conjugate the binding peptide specifically at the tips of the microneedles [21]. Therefore, prior to introducing NOA 63 into the mold, the Au NRs were positioned at the tip of each microneedle. Following this, the mold, now containing Au NRs, was subjected to vacuum to facilitate the filling process. Since the Au NRs were suspended in water, the mixture was heated at 70 °C until the water phase evaporated, ensuring the Au NRs remained affixed to the microneedle tips.

### 2.4. Microplastic Characterization and Detection (FT-IR, Raman, FE-SEM)

The Fourier transform infrared (FT-IR) spectra of the microplastic samples were acquired using an Alpha II spectrometer (Bruker, Billerica, MA, USA) to confirm their surface chemical characteristics. For this analysis, the baseline was established using a blank surface, devoid of any sample at the measurement location.

The morphology of the microneedle and the microplastics adhered to it was verified through field-emission scanning electron microscopy (FE-SEM) analysis. To prepare for this examination, the microneedle, after being amalgamated with the peptide and microplastic, was dried at 25 °C overnight. Subsequently, the dried sample was affixed to a wafer with carbon tape and coated with platinum for 120 s under a 3 mA current. The FE-SEM images were captured using a SIGMA 300 microscope (Carl Zeiss, Weimar, Germany).

Raman spectroscopy measurements for the microplastics, both in isolated states and when bound to microneedles, were conducted using a commercial Raman instrument (XperRam S, Nanobase Inc., Seoul, Republic of Korea). The spectra were collected employing a 532 nm laser as the excitation source, with an incident power set to 2 mW. The laser beam was focused onto the samples using 10× and 40× objective lenses (MPlanFLN, Olympus, Tokyo, Japan), while gratings of 600/600 lpmm were utilized to disperse light within the spectrometer (XPE 200, Nanobase Inc.) during the Raman experiments. For milling particle analysis, the exposure time was configured to 2000 ms. In contrast, for samples simulating environmental conditions, five measurements were conducted at 500 ms each, employing an averaging mapping technique to enhance data reliability.

### 2.5. Microplastic and Peptide Binding Test

Given the hydrophobic nature of the microplastics targeted for detection, a hydrophobic plastic-binding peptide was selected for this purpose. Furthermore, peptides previously utilized in research—namely PSBP, PPBP, and PEBP—were employed for their established efficacy. For the verification of fluorescence, peptides labeled with FITC at each terminus were used. The peptide solutions were prepared at a concentration of 200 μg/mL in deionized (DI) water. To each glass vial, approximately 1 mg of microplastics was added, followed by 500 μL of the peptide solution. The vial was placed in a multimixer (SLRM-3; SeouLin Bioscience, Seongnam, Gyeonggi-do, Republic of Korea) and spun in F1 mode at 35 rpm for 30 min to facilitate the binding between the plastics and peptides. After 30 min, the supernatant was removed as completely as possible, and the particles that remained were recovered for analysis using a fluorescence microscope.

To attach microplastics to microneedles via peptides, peptides with termini modified by 3-Mercaptopropanal groups were initially incubated on microneedles for 30 min at a concentration of 200 μg/mL. Following incubation, the microneedles were washed, and three different types of microplastics, each at a concentration of 0.5 mg/mL, were subsequently incubated on the microneedles for another 30 min. Throughout these procedures, the microneedles were positioned upside down in the solution to ensure an effective reaction. After the incubation, the microneedles were washed in the same manner, dried at room temperature, and prepared for subsequent analysis.

### 2.6. Animal Care and PE Feeding

BALB/c Cr Slc mice, aged 6 weeks, were sourced from the Shizuoka Laboratory Center (Shizuoka, Japan) and maintained in temperature-controlled clean racks under a 12 h light/dark cycle. For the experimental procedures, polyethylene (PE) was dissolved in Tween-80 (Sigma-Aldrich, USA) to prepare a 10% weight/volume stock solution. This stock solution was subsequently homogenized using a Q-Sonica homogenizer (Newtown, PA, USA). The mice were orally administered a daily dose of 100 ppm/100 μL of PE. All procedures involving animals were performed in strict accordance with the guidelines set forth by the Korea Institute of Radiology and Medical Sciences (KIRAMS) and were approved by the Institutional Animal Care and Use Committee (IACUC number: KIRAMS 2023-0022).

### 2.7. Organ Lysis

Following a four-week period of PE administration, mice (n = 5) were euthanized using CO_2_. To determine the concentration of PE in the small intestine, HCl (5 mL, 30% *v*/*v*) was applied to the tissue at room temperature for a duration of three days.

### 2.8. Microplastic Pretreatment in Small Intestine

After neutralizing the tissue solution with NaOH, dialysis was performed to remove the resulting NaCl. The neutralized solution was encased in a dialysis bag and incubated in DI water for 24 h. Post-dialysis, the solution was collected, and 30% H_2_O_2_ was introduced to degrade organic matter. To this end, 3 mL of 30% H_2_O_2_ was added to 10 mL of the dialyzed solution, and the mixture was incubated at 60 °C for 24 h. Subsequently, the solution underwent vacuum filtration through a membrane with a pore size of 0.22 μm. The filtrate was subsequently washed with DI water, and the material on the membrane was recovered with a small volume of DI water for concentration.

## 3. Results

### 3.1. Characterization of Microplastic Particles

**Figure 2:**
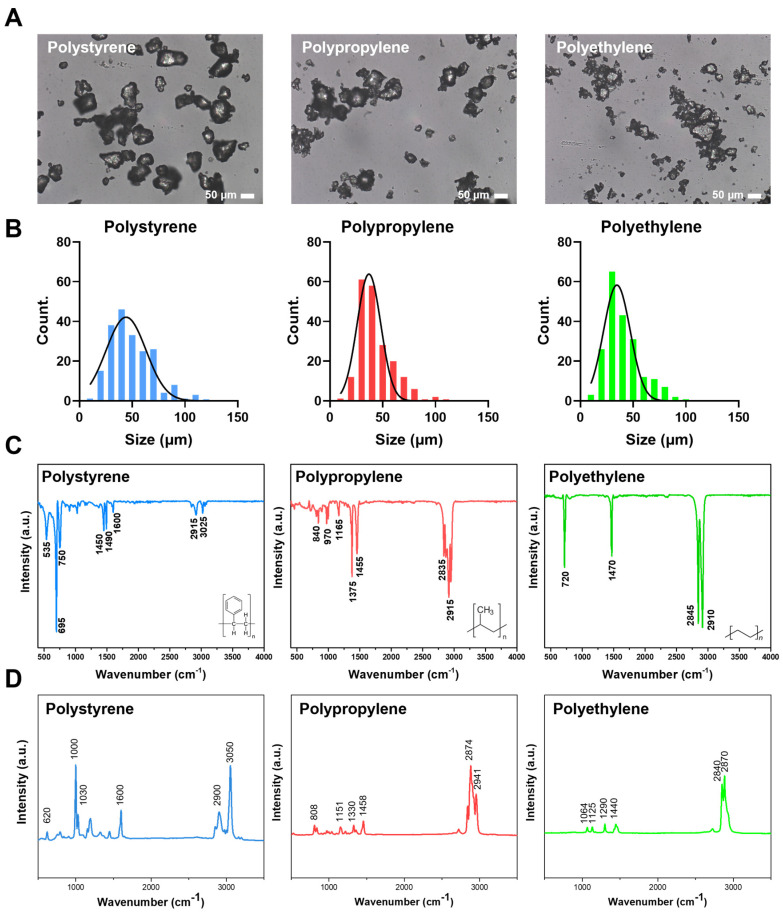
Characterization of microplastics using multiple techniques. From left to right, the analysis results of three targeted microplastics, polystyrene, polypropylene, and polyethylene, are shown. In (**A**), the size and morphology of the microplastics were measured through micrographs and their image analysis was performed. In (**B**), the results of the image analysis show the particle size distribution, and the average size and distribution differences of the particles can be seen. (**C**) FT-IR spectral measurements, each of which shows a specific chemical bond peak, thus confirming the nature of the particles. (**D**) Raman microscopy is utilized to confirm the presence of the target microparticles, which can be seen in conjunction with the FT-IR spectra results.

Three types of plastics in granular form were pulverized using a blender to obtain a powdered form. Subsequently, particles within the size range of 38 μm to 53 μm were selected and characterized. The morphology and dimensions of the resultant microplastic particles were examined under a microscope (Figure 2A), with the actual sizes being quantified via ImageJ software. The size frequency distribution illustrated that all three types of microplastics fell within 38–53 μm (Figure 2B).

Furthermore, the chemical attributes of these microplastic particles were analyzed via FT-IR. In the case of PS, notable absorptions included C–H stretching vibrations at 3025 cm^−1^, methylene groups at 2915 cm^−1^, and C=C stretching vibrations at 1600 cm^−1^, 1490 cm^−1^, and 1450 cm^−1^ [22]. PP was characterized by functional groups indicative of C–H bonds at 840 cm^−1^, C–C bonds at 970 cm^−1^, and CH_3_ groups at 1375 cm^−1^ and 1455 cm^−1^ [23]. In the case of PE, the analysis confirmed the presence of CH_2_ asymmetric C–H stretching at 2910 cm^−1^, CH_2_ symmetric C–H stretching at 2845 cm^−1^, and CH_2_ bending vibrations at 720 cm^−1^ [24] (Figure 2C).

Raman spectroscopy further elucidated the molecular characteristics of each plastic type (Figure 2D). PS displayed peaks at 1000 cm^−1^ and 3050 cm^−1^, indicative of aromatic ring structures [25]. PP exhibited peaks at 808 cm^−1^ and 1458 cm^−1^, corresponding to C–C bonds and CH_2_ groups, respectively [26], with the presence of CH_3_ groups being confirmed by a peak at 2874 cm^−1^ [27]. PE was characterized by peaks at 1125 cm^−1^, 1290 cm^−1^, 1440 cm^−1^, 2840 cm^−1^, and 2870 cm^−1^, which were attributed to the polyethylene C–C bond and CH_2_ groups [28]. These findings confirmed the consistency of the microplastic properties pre- and post-grinding.

### 3.2. Microplastic and Peptide Binding Test

**Figure 3:**
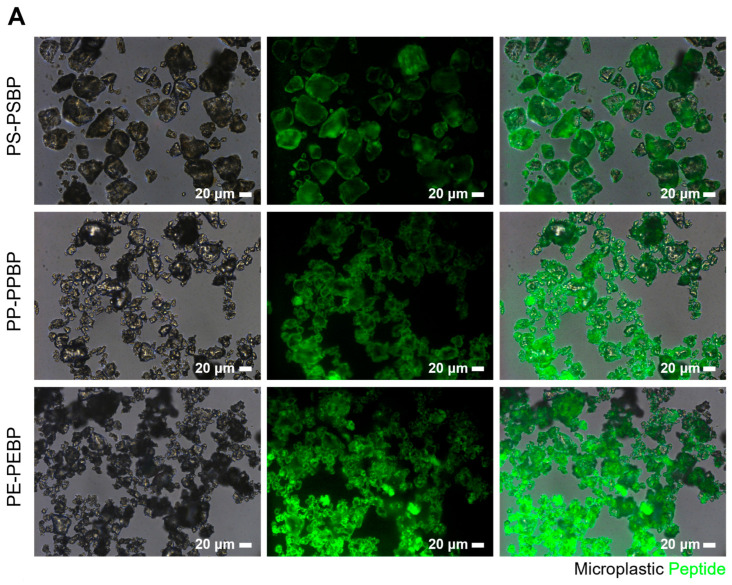

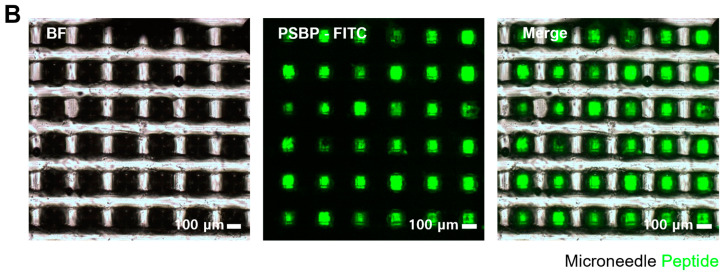
Microplastics and peptide binding assay. (**A**) Fluorescence image of the binding between peptide modified with FITC at the N-terminus and microplastics. (**B**) Fluorescence image of PSBP-decorated microneedles. PSBP is modified with SH groups and FITC at the terminal ends, and Au NRs are encapsulated at the tip ends of the microneedles. The microneedles were photographed in a downward direction from top to bottom. BF indicates bright field.

To confirm the binding of peptides to microplastics, FITC-labeled peptides were employed. PS, PP, and PE are all hydrophobic molecules made up of carbon and hydrogen atoms, and they are nonpolar. Similarly, peptides also contain hydrophobic parts made up of amino acids. These nonpolar parts tend to repel each other or stick together when they are in close proximity to each other. In the case of PS in particular, the presence of an aromatic ring allows for a π-π interaction with the amino acids Histidine (H) and Tryptophan (P), and others that have this structure. This is known as a hydrophobic interaction [29]. We demonstrated in previous studies that microplastics could bind to peptides through hydrophobic interactions at concentrations as low as 0.5 μg/mL, and the occurrence of binding was observed even at lower peptide concentrations. For this experiment, 200 μg/mL of PSBP, PPBP, and PEBP, each fluorescently labeled as per the methodology outlined in the reference, were introduced to the microplastics and subsequently analyzed through fluorescence imaging. The imaging results indicated that the peptides uniformly adhered to all three types of microplastics (Figure 3A).

Microneedles served as a selective sensor for the detection of microplastics. The structure of the microneedle is designed to capture plastics as small as 5 mm, which are difficult to see with the naked eye. It is also highly hydrophobic, which aids in the detection of microplastics, and promotes the evaporation of sample solutions containing water, facilitating analysis. By manipulating the movement and rotation of the microneedle substrate, the Raman laser beam and microplastics can be easily brought into close contact, which is effective for microplastics detection. To facilitate the binding of peptides to the microneedles and consequently capture microplastics, the microneedles were engineered with Au NRs at their tips, and a 3-Mercaptopropanal group was appended to the peptide termini to promote binding between the SH groups and gold. Utilizing the same FITC-labeled peptides as in the microplastic binding assay, their binding to the microneedles was examined under fluorescence microscopy. Fluorescence was notably observed in the microtip area (Figure 3B), confirming the specific attachment of the peptide to the gold nanorods at the microneedle tips. This outcome highlights the potential for effective binding between the microneedle and microplastics.

### 3.3. Peptide-Mediated Microplastic Binding in Microneedles

**Figure 4:**
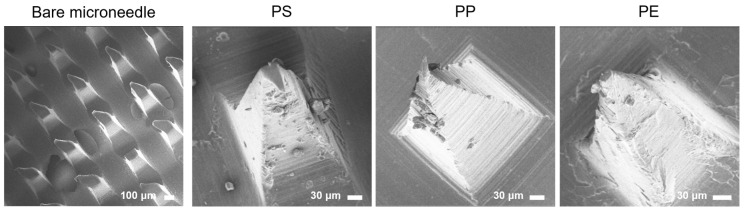
Microneedle and microplastic binding by peptide. SEM image of microneedles with microplastics combined using specific peptides.

Following the successful demonstration of peptide binding to three distinct microplastics and the microneedle tip, the next objective was to validate the peptide-mediated detection of microplastics on the microneedle tip. Peptides were affixed to the microneedles for 30 min, after which, the microplastics were exposed to the peptides for an additional 30 min to facilitate binding. Each step was followed by a washing procedure to eliminate any unbound peptides and microplastic particles. The binding of microplastic particles to the microneedle tips was visually confirmed via SEM. The SEM images revealed the adherence of microplastics to the microneedle tips (Figure 4). These findings confirm the occurrence of peptide binding to the microneedle tips and that this binding was selective, driven by the hydrophobic interactions between the microplastics and the peptides. Moreover, the results indicate that the attachment of the microneedle to the peptides, which presented exposed SH groups, and the subsequent binding of these peptides to the microplastics, occurred efficiently within a relatively short timeframe and was sustained over an extended period. However, while SEM analysis provides a means to visually detect the presence of microplastics, it falls short in identifying the specific material properties of the bound particles. To overcome this limitation and analyze the structural characteristics of the attached particles more comprehensively, Raman spectroscopy was proposed as the next analytical step.

### 3.4. Detection of Microplastics by Raman Spectroscopy

**Figure 5:**
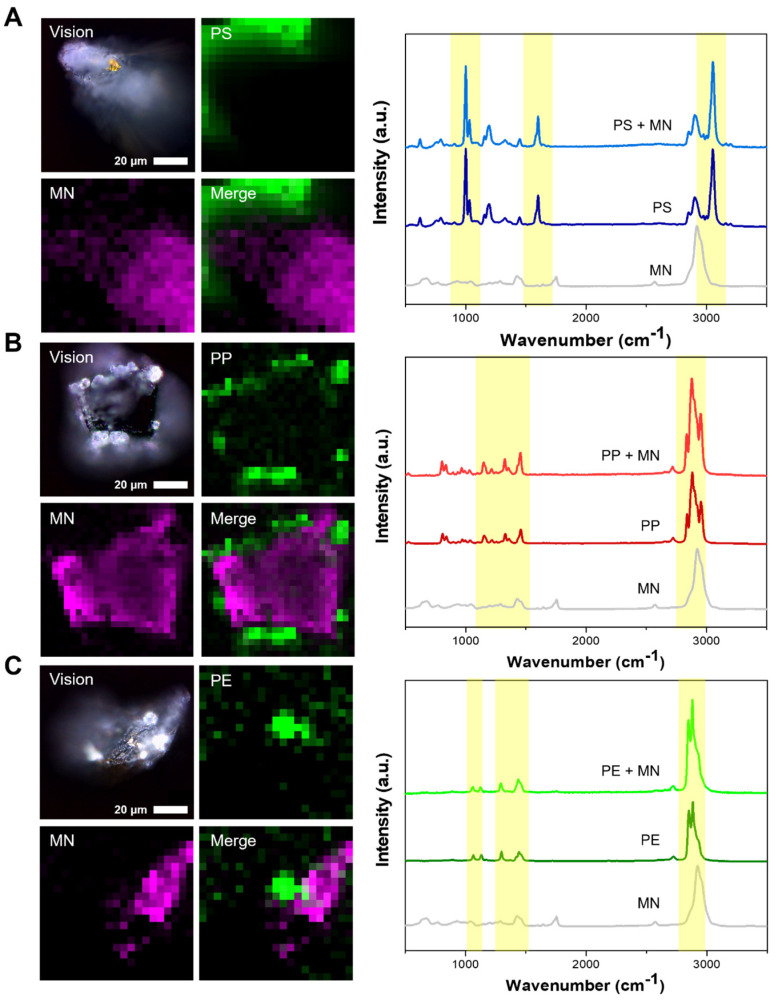
Detection of microplastics by Raman spectroscopy. From above: PS, PP, and PE bounded to microneedle. (**A**–**C**) Raman signal detection of microplastics using 3D mapping. MN stands for microneedle.

Raman spectroscopy was employed to characterize the microplastics that were attached to the microneedles. For this analysis, two microneedles with bound microplastics were subjected to examination using a 532 nm laser for Raman spectroscopy. Given that the microneedles themselves exhibit a unique spectral peak attributable to the NOA63 material used in their construction, a measurement of a bare microneedle was also conducted for comparison. The analysis revealed that the microplastics affixed to the tips of the microneedles were detectable, displaying spectra that were nearly identical to those of the conventional powder form of the microplastics (Figure 5A–C).

This study’s utilization of microneedles, in conjunction with specific peptides, successfully facilitated the capture of microplastics across various sizes. Characterization through Raman spectroscopy unequivocally confirmed the presence of bound microplastics. The employment of peptides, capable of rapidly binding to microplastics, alongside the innovative use of microneedles, highlights the potential of deploying such sensors for the selective detection of microplastics, even in mixtures containing other materials.

### 3.5. Detection of Microplastics in Simulated Environmental Samples

**Figure 6:**
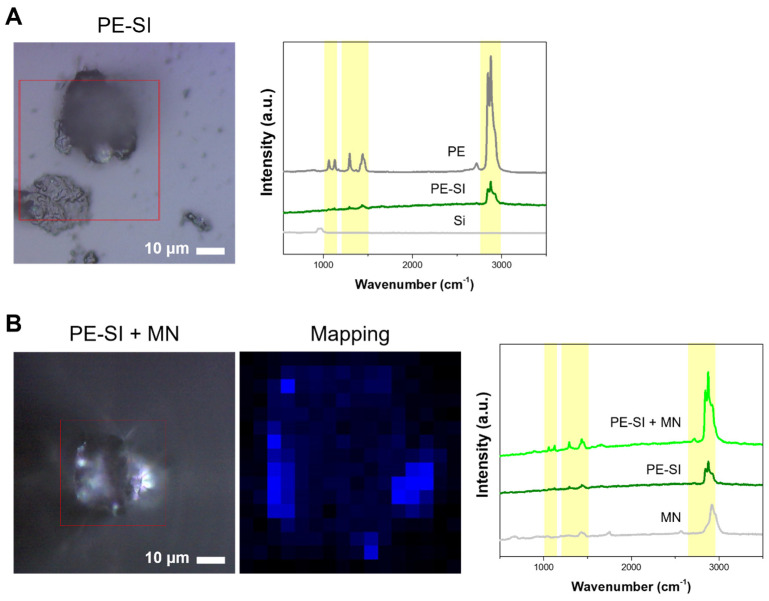
Detection of microplastics in simulated environmental samples. (**A**) Raman signal of PE detected in small intestine samples. (**B**) PE Raman signal in small intestine sample bound to microneedle. PE-SI stands for polyethylene in small intestine, and Si stands for glass.

To further validate our findings and confirm that the developed microneedle functions effectively as a peptide-mediated sensor for the selective detection of microplastics in real-world scenarios, environmental simulation samples were prepared. Given the pervasive nature of microplastics in environments such as oceans and the atmosphere, which can lead to their accumulation in the human gastrointestinal tract via inhalation or ingestion [30], the small intestine of mice that had ingested PE microplastics was chosen for this simulation. The process intended to mimic the accumulation and analyze it under conditions resembling those of the actual environment. The small intestine, harvested from mice administered orally with PE particles sized 3–16 μm, was dissolved in HCl and subsequently neutralized to a pH of 7 using NaOH. To mitigate interference in subsequent analyses from NaCl and organic components such as large tissues present in the solution, dialysis and H_2_O_2_ treatment were employed to eliminate NaCl and oxidatively degrade organic matter [31]. Following filtration to remove the solution, the residual material was concentrated, and the supernatant was separated by exploiting the density difference to settle the larger organic particles. To ascertain the presence of microplastics in this solution, droplets were placed on glass and dried for Raman spectroscopic analysis. This analysis revealed PE’s spectral signature amidst the remaining organic matter, displaying a spectrum akin to that of PE powder (Figure 6). These findings validate the presence of microplastics accumulated in the small intestine, demonstrating they can be analyzed through an appropriate pretreatment.

Subsequent experiments focused on determining whether the microneedle could selectively bind and detect microplastics within this solution. Following the established protocol, the microneedles were incubated in the solution, and microscopic examination revealed particles attached to the microneedle tip, notably devoid of any other organic substances. Raman spectroscopy further confirmed the distinct peak characteristic of PE particles, affirming the microneedle’s capability for selective microplastic capture even in solutions laden with organic materials from various environmental contexts. Thus, the study’s outcomes illustrate that the specific binding peptide bound to the microneedle can effectively capture microplastics in turbid solutions, simplifying the analysis process.

## 4. Conclusions

A microneedle sensor, capable of capturing microplastics via peptides that exhibit specificity toward PS, PP, and PE microplastics, has been developed. The peptides selected for this purpose demonstrated an ability to bind uniquely to the targeted microplastics, with the binding reaction to either the microneedle or microplastics concluding within 30 min. This method facilitated the capture of microplastics of diverse shapes and sizes directly on the microneedles without necessitating an independent recovery technique. This technology has proven its ability to selectively detect microplastics amidst contaminants in environmental simulation samples, positioning the sensor as a practical and user-friendly tool for field applications under real environmental conditions. The innovation in microplastic detection presented herein offers a targeted approach to addressing a critical global environmental issue. The outcomes of this research indicate that the peptide- and microneedle-based microplastic detection method is promising, not only for the exploration of oceanic plastics, but also for the detection of microplastics across various settings and potentially for the recognition of microplastics accumulated within the human body. Consequently, the developed sensor promises an accurate and cost-effective detection of microplastics in diverse environments, contributing significantly to efforts aimed at managing microplastic pollution and mitigating its health and ecological impacts.

## Data Availability

The raw data supporting the conclusions of this article will be made available by the authors on request.
